# Possible Role of Large Fluid Intake in Delaying Formation of Encrustations and, thereby, Prolonging Working Life of Memokath Stent for Nearly 14 Years in a Spinal Cord Injury Patient

**DOI:** 10.1100/tsw.2007.280

**Published:** 2007-10-12

**Authors:** Subramanian Vaidyanathan, Bakul M. Soni, Peter L. Hughes, Gupreet Singh

**Affiliations:** ^1^ Regional Spinal Injuries Centre, District General Hospital, Southport, Merseyside PR8 6PN, UK; ^2^ Department of Radiology, District General Hospital, Southport, Merseyside PR8 6PN, UK

**Keywords:** spinal cord injury, autonomic dysreflexia, Memokath, stent, urinary bladder

## Abstract

The Memokath stent has been used in spinal cord injury patients as a reversible alternative to external urethral sphincterotomy, but the stent has a finite lifetime of <2 years before failure in the majority of patients. We report an unusual case of a spinal cord injury patient in whom memokath stent was functioning for almost 14 years. The long life span of the Memokath in this patient was probably due to this person's habit of drinking around 5 l of fluids a day. Large fluid intake resulted in high urine output and, consequently, deceased the risk of urine infections and delayed formation of encrustations around the stent. Although this case represents an unusual length of time for a Memokath stent to have been in place and functioning, caution should be exercised against the long-term use of Memokath stents. Memokath stents do not get absorbed into the mucosa unlike urolume stents and, therefore, are prone to stone formation. Further, Memokath stents have not yet been approved in the U.S. either for bladder outlet obstruction or detrusor-sphincter dyssynergia. This case is also a reminder to health professionals that if a tetraplegic patient, in whom a Memokath stent has been deployed for treatment of detrusor-sphincter dyssynergia, presents with autonomic dysreflexia, encrustations blocking the lumen of the stent or calculus formation around the stent should be considered as possible reasons for autonomic dysreflexia.

## BACKGROUND

The Memokath stent has a finite lifetime before failure. When Memokath stents were used for bladder outlet obstruction in men unable to undergo transurethral resection of prostate, there were several patients in whom the stent remained in place after 6 years. The Kaplan-Meier survival curve implies that a greater percentage of men die with their stents than outlive the usefulness of the stent, suggesting that the stents are generally well tolerated and have a reasonable life expectancy. Less than a quarter (23%) of stents were considered to have failed the patient[1]. In contrast to the long working life of a Memokath stent when used to relieve bladder outflow obstruction in men with enlarged prostate, the durability of the Memokath stent where the stent is deployed to treat detrusor-sphincter dyssynergia in patients with spinal cord injury appears to be relatively short. A review of 29 patients with spinal cord injury (17 tetraplegic and 12 paraplegic) who underwent stenting of the external urethral sphincter either for prevention of dysreflexic symptoms, high residual urine volumes, and subsequent urinary tract infection or for protection of the upper tracts in the Spinal Injuries Unit, Sheffield, England, revealed that the overall mean working life of the Memokath was only 21 months[2]. Similar findings were reported from the Spinal Injuries Unit, Royal National Orthopaedic Hospital, Stanmore, Middlesex, London, where 25 patients with spinal cord injury underwent Memokath stent insertion, but the majority of stents were removed within 2 years of insertion[3]. In Royal Perth Rehabilitation Hospital, Perth, Western Australia, 26 stents were inserted in 24 patients to combat the development of detrusor-sphincter dyssynergia after spinal cord injury. Removal of the stent became necessary in 19 patients between 2 and 18 months after insertion, with a mean interval of 7 months[4].

We report an unusual case of a spinal cord injury patient who had made it a habit to drink around 5 l of fluid a day, which resulted in large urine output and, consequently, delayed formation of encrustations/stone over Memokath stent for nearly 14 years.

## CASE REPORT

A 15-year-old boy fell from a tree, landing on the back of his head, in 1991. He sustained burst fracture of C-6 and tetraplegia. Trial of micturition was unsuccessful; therefore, he was discharged home with long-term indwelling urethral catheter. In 1993, a Memokath 028 stent, length: 50 mm, was inserted under fluoroscopic control (Fig. 1). Following placement of the Memokath stent, this patient was able to empty his bladder by reflex voiding without manifesting dysreflexic symptoms such as sweating, headache, or increased spasms while passing urine. Ultrasonography of the urinary bladder showed very little residual urine. In 1994, intravenous urography showed the urethral stent *in situ*; no calcification; normal kidneys, ureters, and bladder.

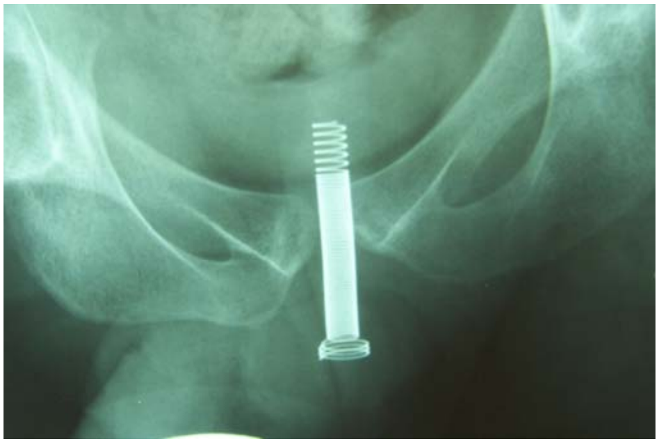

**FIGURE 1.** X-ray of pelvis taken on 20 September 1993 shows good placement of the stent, which is a Memokath 028, 50 mm, and has a distally expanding segment only. For spinal cord injury patients with detrusor-external sphincter dyssynergia, the Memokath 045TW stent is preferable to the Memokath 028. The Memokath 045TW has an expanding segment in both ends of the stent and, therefore, the stent is secured in position by a “double anchor”.

An X-ray of the pelvis taken in 1997 and 1998 showed the urethral stent in good position and there was no calcification. This patient had made it a habit to drink a lot of fluids. On an average, he would drink fluids in the region of 5 l/day. This patient did not get urine infections and was happy with the working of the Memokath stent.

In December 1999, this patient was reviewed with the aim of removing the stent, as the stent had been in place for >6 years. X-ray of the urinary bladder showed no calcification around the stent, therefore, it was decided to defer removal of the stent. In 2002, intravenous urography showed the stent to be in proper place and there was no calcification over the stent; kidneys, ureters, and urinary bladder appeared normal (Fig. 2). During a telephonic interview in April 2007, this patient informed a health professional that he was doing fine.

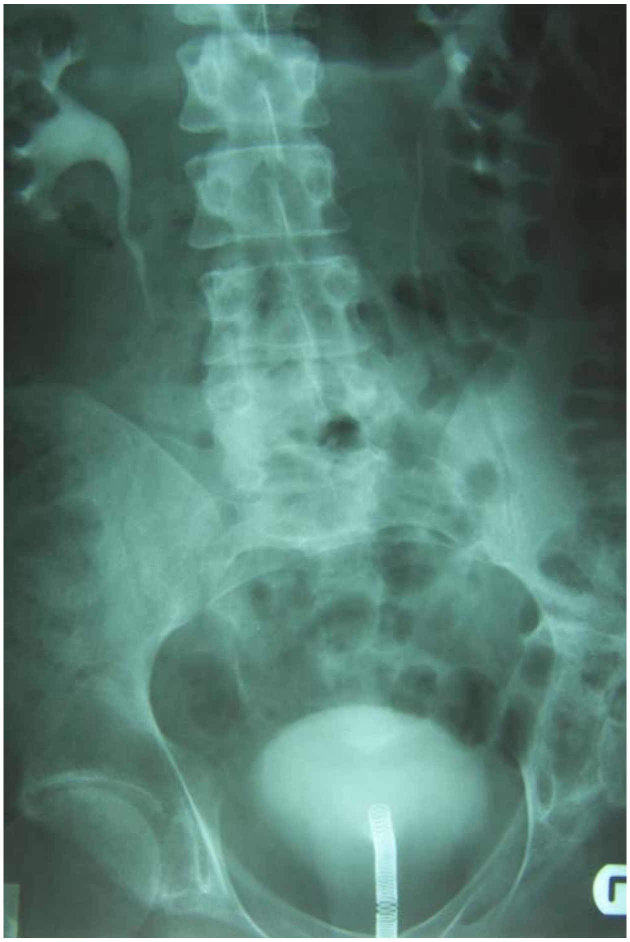

**FIGURE 2.** Intravenous urography (20-min film) performed on 27 August 2002 shows normal upper tracts. There is no migration or calcification of the stent.

In June 2007, this patient required emergency admission to the spinal unit because of chills, rigor, sweating, and headache. He had penile sheath drainage. There was no problem in passing urine. There was no pressure sore. Bowel movement had been satisfactory. Blood tests showed slightly elevated C-reactive protein (25.8 mg/dl; range: 0.0–10.0). White cell count was normal. Plasma sodium was 138 mmol/l (reference range: 133–146 mmol/l). Creatinine: 86 μmol/l (reference range: 0–135 μmol/l). Diagnosis on admission was urinary infection with autonomic dysreflexia. He was prescribed ciprofloxacin and his condition improved. He became afebrile; no longer did he develop autonomic dysreflexia, although he felt sweaty after drinking fluids. Blood culture showed no growth after 72 h of incubation. Urine microbiology report was *Enterococcus faecalis*, which was sensitive to amoxicillin He was discharged home.

Subsequently, X-ray of pelvis showed the Memokath stent in proper place with no sign of migration, but there was a stone around the vesical end of the stent (Fig. 3). Intravenous urography revealed bilateral hydronephrosis and hydroureter (Fig. 4). As there was a large stone in the urinary bladder around the stent, the patient was advised open surgery to remove both the stone and Memokath stent. The urinary bladder was exposed by suprapubic transverse incision. The urinary bladder was not distended. A vertical incision was made on the anterior bladder wall, thus exposing the stone around the proximal end of the stent. Sterile 0.9% sodium chloride, which was cooled to around 5$^{o}$C was instilled inside the urinary bladder. A 12 Fr catheter was inserted per urethra into the distal end of the stent and sterile cold saline was irrigated through the catheter. Following irrigation with cold saline, coils of the Memokath stent distal to the stone could be untwisted, and the Memokath stent along with the stone was removed *en bloc* through cystotomy. There was no bleeding. Suprapubic cystostomy was performed. The patient made an uneventful recovery. Examination of the stent showed no tissue in-growth. Apart from a large calculus at the vesical end of the stent, there was concretion occluding the lumen of the stent over a short segment just below the stone (Fig. 5). In September 2007, a Memokath 045TW stent, 70 mm in length, was inserted and the patient resumed reflex voiding without developing headache or sweating.

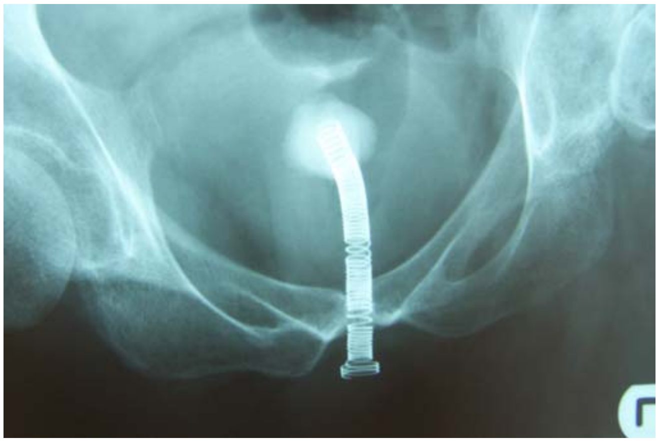

**FIGURE 3.** X-ray of urinary bladder taken on 05 June 2007 shows a stone around the proximal end of the Memokath stent.

## COMMENT

For spinal cord injury patients with detrusor-external sphincter dyssynergia, the Memokath 045TW stent is preferable to the Memokath 028. The Memokath 045TW has an expanding segment in both ends, which spread out to 44 CH when infused with sterile water at 55$^{o}$C and, therefore, the Memokath 045 TW stent is secured in position by a “double anchor”. Further, a long stent, which splints bladder neck, prostatic urethra, and external urethral sphincter, should be deployed in spinal cord injury patients so that the bladder neck as well as the external sphincter is kept open by the stent[5]. For measuring the length of the Memokath stent required for a spinal cord injury patient, the distance between the bladder neck and just beyond the external urethral sphincter should be measured by using two pegs; one peg is kept over the cystoscope when the cystoscope is positioned at the level of the bladder neck, and another peg is kept when the cystoscope is withdrawn distal to the external urethral sphincter. The actual distance is measured by keeping a ruler from *outside* one peg to *inside* the second peg. For measuring the distance,

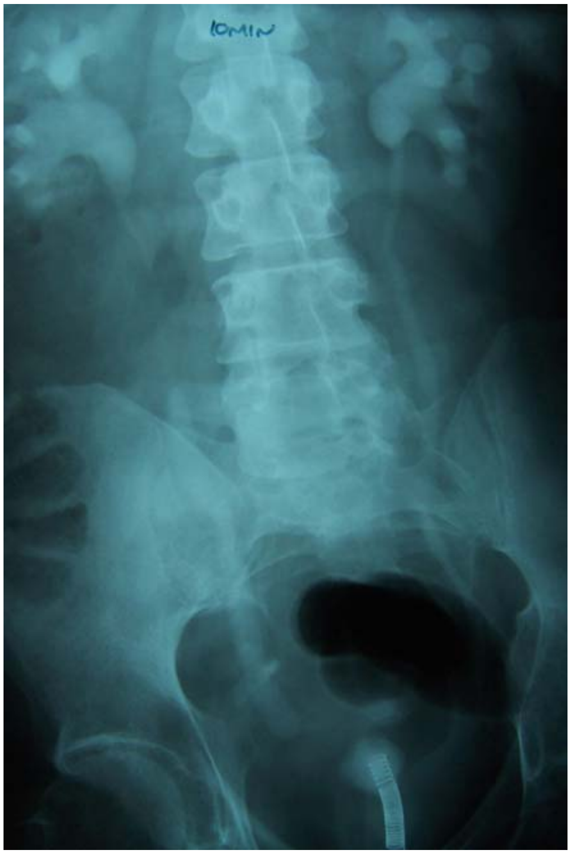

**FIGURE 4.** Intravenous urography (10-min film), performed on 05 June 2007, shows bilateral hydronephrosis and hydroureter.

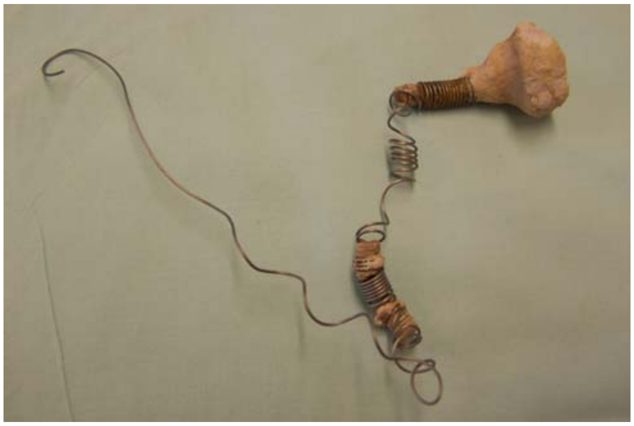

**FIGURE 5.** Photograph of a Memokath 028 stent, after the stent was removed from the urinary bladder by suprapubic cystostomy. There was a large calculus around the proximal end of the stent. In addition to the stone, there was calcification inside the coil of the stent in the mid-segment of the stent. Please note that the stent wire got untwisted in the distal segment when the stent was irrigated with saline cooled to around 5°C, but calcification within the stent in the mid-segment prevented unwinding of the coil. Thus, calcification within the coil of the stent and consequent failure to unwind precluded removal of the stent per urethra, as the stent would not come out as a long twisted wire even after irrigating it with cold saline.

either a flexible cystoscope or a rigid cystoscope can be used. However, the duel-expansion Memokath 045TW can be placed only with the optic lens from a *rigid* cystoscope. The spinal cord physician should be aware that the stent length is measured between the two expansions of the Memokath 045TW. Therefore, while unpacking a new stent, the stent may look longer when its ends have not expanded.

In patients who were treated for benign bladder outflow obstruction by the Memokath stent, encrustation was rare. This was attributed to particular characteristics of the Memokath alloy or the gentle massaging action of subtle movement within the prostatic urethra[1]. Kehinde and associates[6] studied factors affecting the fate of prolonged forgotten “J” stents. Two adult patients with moderate renal failure and producing hypotonic urine retained “J” stents for 40 and 60 months, respectively. Both stents were removed intact endoscopically, and showed no sign of fracture or calcification. Thus, large urine output appeared to confer protection against encrustation of ureteric stents. We may surmise that intake of large quantities of fluids and, consequently, passage of a large quantity of urine will confer similar protection against encrustation for urethral stents as well.

In persons with spinal cord injury, encrustation of the Memokath stent appears to be a problem. Low and McRae[4] reported an average time of 13 months (range: 5–18 months) for development of encrustation around the Memokath stent in spinal cord injury patients. Our patient had been drinking around 5 l of fluids a day and, consequently, was passing large quantities of urine. Large urine output (>3 l in 24 h) delayed formation of encrustations over the stent for more than a decade in our patient. An increase in fluid intake is routinely recommended for patients who have had nephrolithiasis to decrease the likelihood of recurrence of kidney stones[7]. Therefore, it is logical to conclude that drinking large quantities of fluids played a pivotal role in delaying formation of encrustations over the Memokath stent in our patient. Polydipsia can produce hyponatraemia; sodium concentration of 118–127 mmol/l was reported in five patients with epilepsy and polydipsia[8]. In our patient, we were conscious of this possible side effect of drinking large quantities of fluids, as tetraplegic subjects are vulnerable to develop hyponatraemia. Therefore, we performed blood tests to check sodium levels during follow-up visits. Sodium concentration was 141 mmol/l in December 1998, 139 mmol/l in August 2002, and 138 mmol/l in June 2007. Thus, our patient was able to maintain plasma sodium within the reference range. Another benefit of increased consumption of a variety of fluids, including water, may be a significant reduction in the risk of bladder cancer by reducing the overall impact time of potential carcinogens on bladder tissue[9]. This may be pertinent, especially to persons with spinal cord injury who are at high risk for developing neoplastic changes in the neuropathic bladder[10].

## CONCLUSION

The long life span of the Memokath in this patient was probably due to this person’s habit of drinking around 5 l of fluids a day. Large fluid intake resulted in high urine output and, consequently, deceased the risk of urine infections and delayed formation of encrustations around the stent. Although this case represents an unusual length of time for a Memokath stent to have been in place and functioning, caution should be exercised against the long-term use of Memokath stents. Memokath stents do not get absorbed into the mucosa unlike urolume stents and, therefore, are prone to stone formation. Further, Memokath stents have not yet been approved in the U.S. either for bladder outlet obstruction or detrusor-sphincter dyssynergia. This case is also a reminder to health professionals that if a tetraplegic patient, in whom a Memokath stent has been deployed for treatment of detrusor-sphincter dyssynergia, presents with autonomic dysreflexia, encrustations blocking the lumen of the stent or calculus formation around the stent should be considered as possible reasons for autonomic dysreflexia.

## References

1. Perry, M.J., Roodhouse, A.J., Gidlow, A.B., Spicer, T.G., and Ellis, B.W. (2002) Thermo-expandable intraprostatic stents in bladder outlet obstruction: an 8-year study. *BJU Int*. **90(3),** 216–223.
2. Mehta, S.S. and Tophill, P.R. (2006) Memokath stents for the treatment of detrusor sphincter dyssynergia (DSD) in men with spinal cord injury: the Princess Royal Spinal Injuries Unit 10-year experience. *Spinal Cord* **44(1),** 1–6.
3. Hamid, R., Arya, M., Wood, S., Patel, H.R., Shah, P.J. (2003) The use of the Memokath stent in the treatment of detrusor sphincter dyssynergia in spinal cord injury patients: a single-centre seven-year experience. *Eur. Urol*.**43(5),** 539–543.
4. Low, A.I. and McRae, P.J. (1998) Use of the Memokath for detrusor-sphincter dyssynergia after spinal cord injury--a cautionary tale. *Spinal Cord* **36(1),** 39–44.
5. Shah, N.C., Foley, S.J., Edhem, I., and Shah, P.J. (1997) Use of Memokath temporary urethral stent in treatment of detrusor-sphincter dyssynergia. *J. Endourol*. **11(6),** 485–488.
6. Kehinde, E.O., Al-Awadi, K.A., Tawheed, A., Al-Hunayan, A., Ali, Y., and Mahmoud, A.H. (2001) Factors affecting the fate of prolonged forgotten 'J' stents. *Scand. J. Urol. Nephrol.* **35(3),** 222–227.
7. Curhan, G.C., Willett, W.C., Speizer, F.E., and Stampfer, M.J. (1998) Beverage use and risk for kidney stones in women. *Ann. Intern. Med*. **128(7),** 534–540.
8. Okazaki, M., Ito, M., and Kato, M. (2007) Effects of polydipsia-hyponatremia on seizures in patients with epilepsy. *Psychiatry Clin. Neurosci*. **61(3),** 330–332.
9. Moyad, M.A. (2003) What do I tell my patients about drinking water and the risk of bladder cancer? *Urol. Nurs*.**23(5),** 371–377.
10. Vaidyanathan, S., Parsons, K.F., Krishnan, K.R., Soni, B.M., Singh, G., and Sett, P. (1999) What is the optimum fluid intake in male patients with spinal cord injury and neuropathic bladder? *Spinal Cord* **37,** 594–595.
